# Ten important roles for academic leaders to promote equity, diversity, and inclusion in data science

**DOI:** 10.1186/s13040-021-00256-9

**Published:** 2021-03-30

**Authors:** Jason H. Moore, Van Q. Truong, Ashley B. Robbins, David Nicholson, Clar Lynda Williams-Devane

**Affiliations:** 1 Institute for Biomedical Informatics, Perelman School of Medicine, University of Pennsylvania, Philadelphia, PA USA; 2 Genomics and Computational Biology Graduate Program, Perelman School of Medicine, University of Pennsylvania, Philadelphia, PA USA; 3 Neuroscience Graduate Program, Perelman School of Medicine, University of Pennsylvania, Philadelphia, PA USA; 4 Department of Mathematics and Computer Science, Fisk University, Nashville, TN USA

Data science is by design disciplinarily diverse drawing from applied mathematics, computer science, informatics, statistics, and intersecting domains such as biology and medicine. This makes it an exciting and fast moving discipline. Equally important to the success of data science is a diversity of experiences and perspectives which come from people of different backgrounds. To grow and maintain a diverse workforce in data science, leaders must work to create and maintain a representative community of individuals from diverse backgrounds. We review here ten important roles for academic leaders to promote, support, and advance equity, diversity, and inclusion (EDI) throughout data science. No single leader can exemplify every characteristic outlined below, but in order to advance EDI principles in the field, it is our duty to operate in conjunction with each other to fulfill as many characteristics as possible. This work may involve fulfilling multiple roles or creating a support network which works together to chip away at inequality and inequity throughout data science. Here, equity is intentionally placed first in the conversation to emphasize its underlying importance in achieving diversity and inclusion within data science.

## The mentor

There is no doubt that many leaders would not be in a major leadership position without a long series of outstanding mentors going back to high school, but not everyone has that same privilege. Academia is complex and many of the lessons and skills necessary for success come with many years of experience, which are not realized by everyone. The role of a mentor is to pass along the wisdom which comes from a lifetime of success and, importantly, failure. Some academic skills are like the mathematical tricks which make calculus accessible. Without help one might learn some of the calculus tricks and shortcuts, but this takes time and it might be impossible to learn all of the necessary tactics. Equally important are the soft skills mentors can imbue in their trainees. No one can do science alone, but the mentor can cultivate a relationship and environment where the trainee learns to identify their strengths and weaknesses, seek help, work with others, and communicate data science effectively. Data science can be particularly challenging in academia because it is new and few understand what it is and why it is important. This makes mentoring in this discipline even more important, especially for retaining, promoting, and empowering those from underrepresented backgrounds. It is important that mentors avoid conscious and unconscious microaggressions where they edit out or erase the diverse perspective, career path, or vision of their diverse trainee or junior colleague. It is not the role of the mentor to create a carbon copy of themselves, but rather to nurture the uniqueness of their trainees or junior colleagues.

## The champion

Leaders are expected to be good mentors to all students, staff, and faculty. However, there is sometimes the need to go the extra mile for a particular person who you believe in, but who needs an extra time commitment for success. Leaders are usually unable to make these kinds of commitments to more than a handful of people due to their limited availability related to the demands of being a dean, chair, or director. Because of the extreme positive influence a champion can have on a trainee’s career, it is important to allocate this time with an eye toward helping those from disadvantaged backgrounds access opportunities in order to help them achieve equity in an uneven playing field. Data science could be particularly challenging because it is a blend of different disciplines each of which brings its own cultural nuances. A good leader should be familiar with the cultural differences of applied mathematics, computer science, informatics, information technology, statistics, and specific intersecting domains such as biology, economics, or medicine. Being a true champion for your trainee requires insight into their needs and goals, but also the wisdom and desire to provide guidance on how to navigate the disciplinary terrain of their choice. Champions do not think about their own gains in these moments, but are instead concentrated on the personal and professional growth of their trainees. However, even with the best of intentions this may not be possible in the traditional manner. Alternatively, a champion will know how to form the right support team for their student, staff, or faculty member. A champion must exhibit a commitment to leveling the playing field in meaningful ways such as providing a career or leadership coach. The diversity of data science in combination with a diverse background can take additional resources beyond what is possible of one person. A champion can be the captain of the resource team.

## The exemplar

Some of the most respected and admired leaders are the ones who lead by example. It will be much more effective to be an advocate and referee if it is clear you are practicing the principles of EDI in your own research lab and/or academic unit. Start by ensuring you are recruiting students, staff, and faculty from diverse backgrounds. This by nature requires extra effort due to hiring structures which have been historically biased. However, it is necessary and will go a long way toward the broader EDI objectives which come with an academic leadership position. Traditional metrics of success do not recognize the inequitable reality which diverse students, staff, and faculty face. Thus, it is important to acknowledge throughout the hiring process the labor which scholars from underrepresented backgrounds perform outside of their formal duties to make the workplace a more equitable and inclusive environment for everyone. An effective leader must attribute value to these activities to create an equitable hiring process in recognition of the value added to a department, program, or school by diverse perspectives and representatives. Additionally, diverse workplaces alone do not create safe and inclusive places. Exemplary leaders must work to cultivate an environment where each member of their lab or unit feels included, heard, and respected by the team as a whole.

## The practitioner

Particularly relevant to data science is algorithmic or methodological fairness. Artificial intelligence and machine learning algorithms are particularly good at finding and exploiting patterns in data. Some of these patterns could be biased in a way that makes their deployment and use in the real world unfairly biased too. We as data scientists should be aware of these issues both from the data point of view and that of the algorithm. It is imperative that we strive for fairness in all of our data science approaches and methods. As a leader, it is important to highlight these issues, adopt equitable practices throughout our work, and to educate the community about how biased algorithms can function unjustly in the real world as evidenced in the work done by the Algorithmic Justice League. Towards this effort it is important to recognize the importance of understanding and identifying biased data science efforts as a noteworthy contribution to the field and not as an aside to traditional data science.

## The listener

All EDI considerations must begin with active listening. One of the most important leadership activities a dean, chair, or director can engage in is listening to a diversity of opinions, ideas, and concerns from the unit’s constituents. This means making time at faculty meetings for open discussion. However, it is imperative that the leader set the tone of inclusion and acceptance of diverse and progressive ideas. The leader may also need to make time for meaningful small group or one-on-one discussions and meetings with junior colleagues and trainees to get updates on what they are hearing and learning from the community. This may include having meaningful dialogues with groups organized to advance EDI at the institution and listening to key stakeholders from the institution and the broader academic community as well. Since not all trainees, staff, or faculty feel comfortable coming forward to share their thoughts and experiences, the listener may need to work with the ombuds to grow trust within each community. Although this role can be difficult and require a significant and thoughtful effort, listening should be a daily part of the leadership routine and is necessary for staying informed in an area which is rapidly evolving. It is also a great way to receive feedback about EDI initiatives and areas for improvement or action. The only way to overcome shortcomings is to listen astutely for where they exist and why they persist.

## The empathizer

In addition to being a good listener it is very important to show empathy for those who have not been included or who do not feel like they have been treated justly. Empathy requires an understanding of the challenges endured by those from disadvantaged or historically marginalized backgrounds. It requires not only the willingness of the leader to put themselves in their faculty, staff, and student’s shoes, but the willingness to do the research to understand the historical perspective of why faculty, staff, and students might have certain feelings. The leader must envision paths to their leadership role that are robust to the roadblocks that faculty, staff, and students might have encountered or may encounter in the future. Being an empathetic leader may start with admitting the fact that you may never fully comprehend the adversity your underrepresented trainees or junior colleagues have gone through. But, that is not where it has to end. Empathetic leadership is an active process which requires the determination to seek understanding over time. Relatability can be gained through stories of shared struggles, how you overcame them, or simply validating the difficulties the other person is facing. Empathy is important not only because it helps the individual in question feel understood, but it also helps the leader develop more emotional intelligence and constructive plans of actions for promoting EDI across the unit and institution.

## The ombuds

An effective leader must be trusted. Trust allows people who have been mistreated to come forward and share their experiences in a confidential manner. This is one of the most important aspects of EDI. A leader should listen, sympathize, and be prepared to be a referee if necessary. Real change towards diversity and inclusivity in the workplace cannot happen if mistreated students, staff, and faculty do not feel comfortable enough to come forward. Many academic institutions employ ombuds, similar to mediators, who serve in a confidential and semi-independent role to work on issues where there may be bias or mistreatment. A good leader should have some of these same skills and knowledge, while ensuring confidentiality, respect, and institutional accountability are upheld throughout the formal ombuds process. The complexity of issues of mistrust and mistreatment in data science necessitate an effective leader with an understanding and knowledge of the field to be a leader in the resolution of such matters.

## The referee

Systemic discrimination and prejudice are unfortunately pervasive throughout academia and there will undoubtedly be both small and large incidents of varying scales to mediate as a leader. The dean, chair, or director should be ready to intervene in each situation in a firm, fair, and transparent manner to ensure everyone involved understands the process. This could involve educating those who have mistreated someone due to stereotype-based threats concerning their race, ethnicity, religious beliefs, sexual orientation, or disability, for example. It could also involve disciplinary action. To counteract institutional discrimination, the leader may need to facilitate implicit and explicit bias training at the community-level. It is crucial to ensure that prejudiced acts do not stem from a widespread source and will not occur again. Leaders should be well-versed on how to handle issues as they arise and ensure that the burden is not placed on the affected party to ameliorate their own discriminatory experiences. Additionally, the leader should connect impacted individuals with institutional resources and support networks to lean on since the pain from mistreatment does not resolve once the referee has stepped in.

## The advocate

Leaders have tremendous influence over students, staff, and faculty by nature of their positions and the respect which comes with them. As such, it is important to publicly support and communicate EDI principles and philosophy as an advocate. This support can start in the form of written communications such as email and newsletters in addition to public-facing websites, blogs, and social media. It should also be communicated in faculty meetings, leadership meetings, and any other professional or social gatherings related to the responsibilities of the position. This effort can come from supporting educational initiatives such as a journal club focused on scientific papers addressing or even neglecting equity. Delivering consistent messaging across venues sends the message that EDI issues are important and should be discussed and addressed as part of the culture of the school, department, or center. It should be clear that discrimination and prejudice will not be tolerated at any level within the community and institution. It is critical to ensure that the institution’s communications and planned course of actions are aligned, while taking into account the needs of the communities it hopes to serve*.* Part of this messaging should include the importance of EDI for advancing data science, which is a discipline that has thrived on a diversity of expertise, ideas, and people which have been historically neglected in practice. A data science leader should be familiar with how and why data science is better and stronger with EDI in order to advance the field for all. To this end, a leader should use their platform to ensure equity in representation without burdening those diverse voices such that they are unable to deliver on intellectual contributions to data science.

## The activist

As academia grapples with systemic and institutional bias, it is more important than ever to make real and lasting changes to a biased system which has been in place for centuries. This occurs when individuals are mistreated and devalued due to social norms and infrastructure which benefit the majority for simply following these rules. The most common forms of institutional bias are racism, sexism, and ableism, but many more exist. Real change is difficult and will depend on allyship and activism. Those who have traditionally held the power need to stand up for the rights of those from disadvantaged backgrounds and fight for change from within the unit and institution as well as in their role as a citizen. This is perhaps the most difficult challenge of a data science leader because it requires leveraging one’s privileges to take risks and unnerve the current foundation to elicit necessary changes for the underrepresented minority. Just as important as serving as a representative for those from historically marginalized communities, it is important to change the perception that those individuals are somehow less than or incomparable. While their background and experiences may be different and not without obstacles; they are valuable, intelligent, and talented. It is imperative that diverse voices and their respective cultural uniqueness be given a platform to be heard by effective leaders. Differences are not a catalyst for change, but rather a celebration of progress. Just because things have always been done a certain way, does not mean they should continue in that manner. Allyship and activism have to be part of the EDI equation. It is the only way real change will happen.

## Data Availability

Not applicable.

